# [Expression of Concern] Transformation of nomifensine using ionizing radiation and exploration of its anticancer effects in MCF-7 cells

**DOI:** 10.3892/etm.2026.13276

**Published:** 2026-08-21

**Authors:** Seong Hee Kang, Dong-Ho Bak, Byung Yeoup Chung, Hyoung-Woo Bai

Exp Ther Med 23:306, 2022; DOI: 10.3892/etm.2022.11235

Subsequently to the publication of the above article, an interested reader drew to the authors’ attention that, concerning the western blot data shown in Fig. 2C on p. 5, it was unexpected that the authors would have detected cleaved caspase-3 and caspase-3 expression in the experiments performed using the MCF-7 cell line, since it has been shown that MCF-7 cells do not express caspase-3 (for example, see the paper by Reiner U. Jänicke and colleagues entitled “MCF-7 breast carcinoma cells do not express caspase-3.” Breast Cancer Research and Treatment, vol. 117.1 (2009); p. 219-221).

The authors have been contacted by the Editorial Office to offer an explanation for these unexpected findings in their study, and we are awaiting their response. Owing to the fact that the Editorial Office has been made aware of potential issues surrounding the scientific integrity of this paper, we are issuing an Expression of Concern to notify readers of these potential problems while the Editorial Office continues to investigate this matter further.

